# DFT studies of hydrocarbon combustion on metal surfaces

**DOI:** 10.1007/s00894-018-3585-z

**Published:** 2018-02-02

**Authors:** Mina Arya, Ali Akbar Mirzaei, Abdol Mahmood Davarpanah, Seyed Masoud Barakati, Hossein Atashi, Abas Mohsenzadeh, Kim Bolton

**Affiliations:** 1 Swedish Centre for Resource Recovery, University of Borås, SE 501-90 Borås, Sweden; 2 Department of Chemistry, University of Sistan and Baluchestan, Zahedan, 98135-674 Iran; 3 Department of Physics, University of Sistan and Baluchestan, Zahedan, 98135-674 Iran; 4 Department of Electrical and Computer Engineering, University of Sistan and Baluchestan, Zahedan, 98135-674 Iran; 5 Department of Chemical Engineering, University of Sistan and Baluchestan, Zahedan, 98135-674 Iran

**Keywords:** Density functional theory, Hydrocarbon combustion, Brønsted-Evans-Polanyi relationship, Catalyst

## Abstract

Catalytic combustion of hydrocarbons is an important technology to produce energy. Compared to conventional flame combustion, the catalyst enables this process to operate at lower temperatures; hence, reducing the energy required for efficient combustion. The reaction and activation energies of direct combustion of hydrocarbons (CH → C + H) on a series of metal surfaces were investigated using density functional theory (DFT). The data obtained for the Ag, Au, Al, Cu, Rh, Pt, and Pd surfaces were used to investigate the validity of the Brønsted-Evans-Polanyi (BEP) and transition state scaling (TSS) relations for this reaction on these surfaces. These relations were found to be valid (R^2^ = 0.94 for the BEP correlation and R^2^ = 1.0 for the TSS correlation) and were therefore used to estimate the energetics of the combustion reaction on Ni, Co, and Fe surfaces. It was found that the estimated transition state and activation energies (E_TS_ = −69.70 eV and E_a_ = 1.20 eV for Ni, E_TS_ = −87.93 eV and E_a_ = 1.08 eV for Co and E_TS_ = −92.45 eV and E_a_ = 0.83 eV for Fe) are in agreement with those obtained by DFT calculations (E_TS_ = −69.98 eV and E_a_ = 1.23 eV for Ni, E_TS_ = −87.88 eV and E_a_ = 1.08 eV for Co and E_TS_ = −92.57 eV and E_a_ = 0.79 eV for Fe). Therefore, these relations can be used to predict energetics of this reaction on these surfaces without doing the time consuming transition state calculations. Also, the calculations show that the activation barrier for CH dissociation decreases in the order Ag ˃ Au ˃ Al ˃ Cu ˃ Pt ˃ Pd ˃ Ni > Co > Rh > Fe.

**Electronic supplementary material:**

The online version of this article (10.1007/s00894-018-3585-z) contains supplementary material, which is available to authorized users.

## Introduction

New and improved technologies that produce energy from sustainable sources are attracting considerable attention from chemical companies and scientists due to increased energy demands, decreases in fossil energy reserves, and the negative impact of fossil fuel based energy on the environment, including climate change [1]. Catalytic combustion of hydrocarbons (CH → C + H [2–4]) is an important technology for energy production. It reduces emissions of pollutants and greenhouse gases compared to conventional flame combustion, and is done at lower temperatures [5–8]. Due to their industrial and commercial advantages, metal catalysts are commonly used for this process, and a wide variety of metal-based materials have therefore been studied by experimentalists and theoreticians [9, 10]. Lee et al. [11, 12] studied the dynamics of the activated dissociative adsorption of CH_4_ on Ni (111) by molecular beam techniques coupled with high-resolution electron energy loss spectroscopy (HREELS). The adsorbed CH_3_ radical and H atom were identified as the products of the dissociative reaction. The existence of the chemisorbed CH, CH_2_, and CH_3_ on Ni (111) has also been reported by Yang et al. using X-ray photoelectron spectroscopy (XPS) and secondary ion mass spectroscopy (SIMS) [13, 14]. Experimental investigations of hydrocarbon oxidation on metal surfaces are challenging due to the complexity of the process, and information at the microscopic scale, e.g., the energy barriers and active adsorption sites, which can be determined theoretically, is useful for understanding the chemical rates and mechanisms [15, 16].

The use of density functional theory (DFT) has become standard in computational catalysis [17]. In a recent theoretical study, Mohsenzadeh et al. [18] used DFT calculations to study hydrocarbon combustion and synthesis on Ni surfaces, and found that the activation and reaction energies of these reactions depend on the surface structure. Inderwildi et al. [19] performed DFT calculations to investigate hydrocarbon combustion and synthesis on noble metal surfaces, and showed that the combustion and synthesis mechanisms were similar on all of the metal surfaces investigated. Li et al. [10] used DFT periodic boundary calculations to study methane decomposition on Ni (100), Ni (111), and Ni (553) surfaces. The adsorption sites and energies of the CH_x_ (x = 0–3) and H species on these surfaces were also studied. The results show that the adsorption of CH_x_ and H species is favored on the less packed surfaces, i.e., the Ni (100) and Ni (553) surfaces. Among all of the adsorbates investigated, the adsorption energy of the carbon atom was the most sensitive to the surface structure.

First principles calculations of catalytic reactions are computationally demanding. Identification of one or a few descriptors that can use a data set to correctly predict new data, such as activation energies, can reduce the number of data that need to be explicitly calculated. This would significantly reduce the demand on computational resources. Examples of such descriptors are the Brønsted-Evans-Polanyi (BEP) and transition state scaling (TSS) relations, which correlate the kinetics with thermodynamics of a chemical reaction. As shown in Eq. 1, the BEP relation assumes that the activation energy of a reaction is linearly dependent on the reaction energy. The TSS relation, shown in Eq. 2, assumes a linear correlation of the transition state (TS) energy with the initial state (IS) energy (the TSS can also be formulated to correlate the TS energy with the final state (FS) energy) [20, 21].

1 $$ {\mathrm{E}}_{\mathrm{a}}=\upalpha \Delta \mathrm{E}+\upbeta $$

2 $$ {\mathrm{E}}_{\mathrm{TS}}={\upalpha}^{\prime }{\mathrm{E}}_{\mathrm{IS}}+{\upbeta}^{\prime } $$

**Fig. 1:**
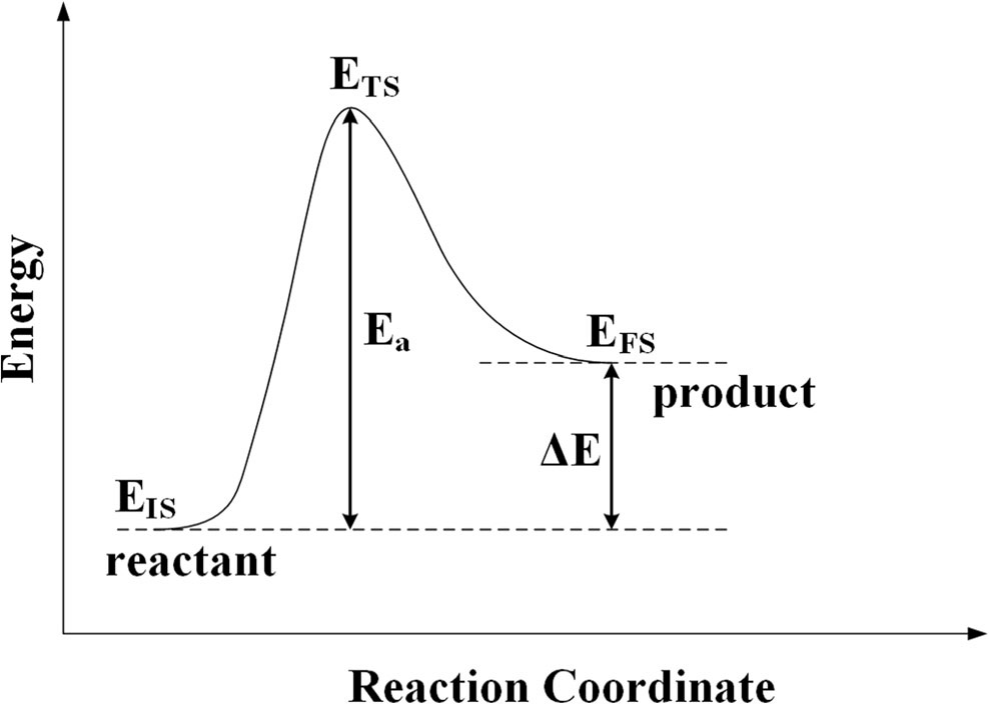
Definitions of the energies used in the BEP and TSS correlations in Eqs. 1 and 2, respectively. The key energies in each elementary reaction are the initial state energy (EIS), the transition state energy (ETS), the final state energy (EFS), the energy of reaction (ΔE), and the activation energy (Ea)

The definitions of the energies used in these equations are shown in Fig. 1.

Fajin et al. [22] used DFT calculations to explore a variety of descriptors, including the BEP relationships, that may be able to predict the catalytic activity of various metallic surfaces for water dissociation. They concluded that the adsorption energy of atomic oxygen on a given metallic surface provides an excellent descriptor of the activation energy for water splitting (H_2_O* + * → OH* + H*, where * is the adsorption site) on that surface.

Wang et al. [20] investigated the ability of the BEP and TSS relations to relate the kinetics and thermodynamics of C−H, C−C, C−O, and O−H bond fission in furan derivatives on Pd (111). They found that the relations perform statistically as well for the furan derivatives as for small C_2_ species. Their results also showed that hydrogenation/dehydrogenation reactions have smaller deviations from the linear BEP and TSS predictions compared to C−O and C−C bond fissions.

This contribution presents results of a comparative and systematic DFT study of hydrocarbon combustion on Ag, Au, Al, Cu, Rh, Pt, and Pd face-centered-cubic (111) surfaces. These metals are used as catalysts in industrial catalytic combustion processes [23]. The calculated energies are used to test the validity of the BEP and TSS for this reaction on these surfaces [24]. Since, as described below, these relations are valid for these systems, they were used to estimate the energetics of this reaction on Ni face-centered-cubic (111), Co hexagonal-closed-packed (111), and Fe body-centered-cubic (111) surfaces. The predicted energies were subsequently compared to data obtained from explicit DFT calculations on these surfaces, using the same methods as used for the previous surfaces. This is the first time that the same models and computational methods are used to investigate catalytic combustion of hydrocarbons on Ag, Au, Al, Cu, Rh, Pt, Pd, Ni, Co, and Fe (111) surfaces to yield insights into the kinetics and mechanisms of this reaction on these surfaces and to test the validity of the BEP and TSS relations for this important catalytic reaction.

This paper is organized as follows: The models and methods used in the DFT calculations are described in the ‘Methods and models’ section, the most stable adsorption sites, reactant, product, and transition state structures and energies are presented in the ‘Results and discussion’ section, along with a discussion of the validity of the BEP and TSS relations for this reaction on these surfaces. There is also a discussion on the relative barrier heights of these surfaces and an attempt to explain this trend using the position of the d-band center and the coordination number of the surface atoms. The activation energies and product and transition state vibrational frequencies are also used to determine the Arrhenius pre-exponential and rate constants at 600 K, which is a typical temperature for industrial catalytic combustion [25–27]. The ‘Conclusions’ section details the most important conclusions of this work, while additional material is given as Supplementary information (SI).

## Methods and models

Spin polarized, generalized-gradient approximation (GGA) DFT calculations were performed using the Vienna ab initio simulation package (VASP) [28–33]. The exchange and correlation energies were calculated using the revised Perdew-Burke-Ernzerhof (RPBE) functional [10, 34–41], and the electron-ion interaction potential in the Kohn-Sham equation was determined using the projector-augmented wave method (PAW) [42].

The parameters used here have been used in previous, successful calculations of similar metal-catalyzed reactions [43–45]. In addition, convergence in the trends presented here has been confirmed by systematically changing the parameters [18] (the size of the k-point mesh, the energy cutoff, the number of metal and vacuum layers used in the model, etc). Briefly, the calculations used a plane-wave basis set with a kinetic energy cutoff of 400 eV [46]. Numerical integration in reciprocal space was performed using the 4 × 4 × 1 Monkhorst–Pack grid of k-points [47], and a dense 41 × 41 × 1 k-point grid was employed for the density of states calculations. A 0.1 eV Fermi smearing was used.

The metal (111) surface was modeled as an infinite periodic slab (in the xy plane) containing three layers of metal atoms with full relaxation of the uppermost two layers and the adsorbates [48]. A (2 × 2) unit cell was used for all calculations. To ensure that the adsorbate does not interact with the neighboring slab, a 10 Å vacuum region was placed between the slabs (in the z direction). All geometries were optimized using a conjugate-gradient algorithm (CG) until the forces acting on each ion and the change in the total energy were converged to less than 10^−3^ eVÅ^−1^ and 10^−5^ eV, respectively [49].

The climbing image-nudged elastic band (CI-NEB) method [16] was used to locate the transition state structures. The reactant and product configurations were used as the initial and final states in the CI-NEB calculations, and a linear interpolation was made to create six images along the elastic band [50]. A -5.0 eVÅ^−2^ spring force constant between images was used to relax all of the images until the force acting on each ion was less than 0.1 eVÅ^−1^.

Vibrational frequencies, approximated as harmonic oscillators, were calculated by diagonalizing the finite difference construction of the Hessian matrix using ionic displacements of 0.01 Å. The frequencies were used to calculate the zero point vibrational energies (ZPVEs) and vibrational partition functions, as well as to ensure that the stationary structures were minimum energy structures (zero imaginary frequencies) or transition states (one imaginary frequency).

The adsorption energies (E_ads_) of the products and reactants were calculated using:

3 $$ {\mathrm{E}}_{\mathrm{ads}}={\mathrm{E}}_{\left(\mathrm{slab}+\mathrm{adsorbate}\right)}\hbox{--} \left({\mathrm{E}}_{\mathrm{slab}}+{\mathrm{E}}_{\mathrm{ads}\mathrm{orbate}}\right) $$

where E_(slab + adsorbate)_, E_slab_, and E_adsorbate_ are the total energies of the slab-adsorbate(s) system, the slab, and the geometry optimized adsorbate(s) in vacuum, respectively [1, 5]. All energies are ZPVE-corrected.

The dissociation rate constants (k) were calculated using transition state theory:

4 $$ \mathrm{k}=\left(\frac{\mathrm{kT}}{\mathrm{h}}\right)\left(\frac{q^{\#}}{q}\right){\mathrm{e}}^{-\frac{\mathrm{Ea}}{\mathrm{kT}}} $$

where k_B_, T, h, and E_a_ are Boltzmann’s constant, absolute temperature, Planck’s constant, and the activation energy, respectively. The activation energy is ZPVE-corrected and q and q^#^ are the partition functions for the initial state and the transition state, respectively [51].

**Fig. 2:**
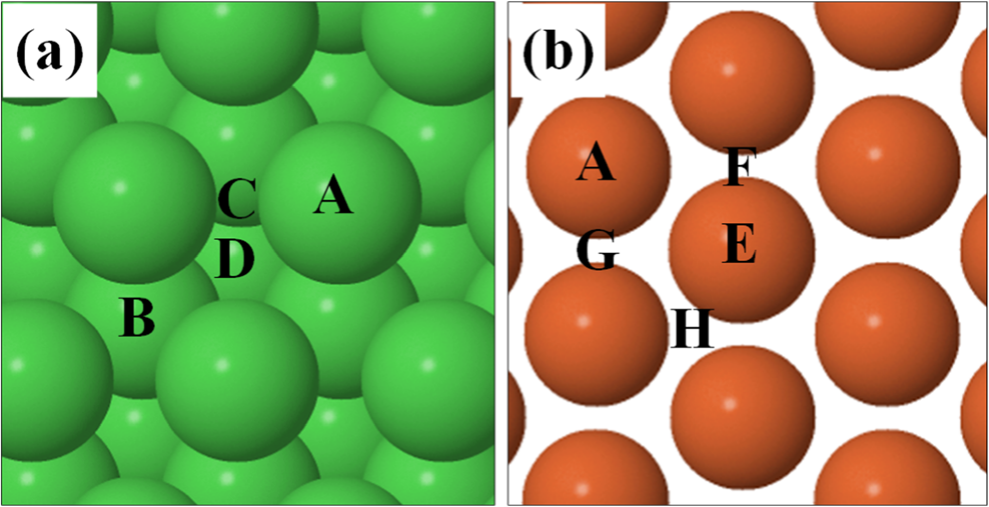
Adsorption sites on the (a) Ag, Au, Al, Cu, Rh, Pt, Pd, Ni, and Co (111) surfaces and (b) Fe (111) surface. A is a top site; B is an hcp site; C is a bridge site; D is a fcc site; E is a rectangular fourfold hollow site; F is a long bridge site; G is a short bridge site and H is a pseudo threefold hollow site

The adsorption sites that are present on the Ag, Au, Al, Cu, Rh, Pt, Pd, Ni, Co, and Fe (111) surfaces and that were investigated are shown in Fig. 2. The adsorption sites are identical for all surfaces (shown in panel a) except Fe (111) (shown in panel b).

The preferred adsorption sites and optimized geometries of adsorbed CH, C, and H were identified by placing these species on each of the adsorption sites shown in Fig. 2, and subsequently optimizing the system to identify the site that yields the lowest ZPVE-corrected energy. The results for CH, which are discussed below with reference to Table 1, yield the optimized reactant adsorption energies (E_IS_ in Fig. 1) and geometries. As described previously [52], the optimized sites, geometries, and energies (E_FS_ in Fig. 1) of the co-adsorbed C + H products were obtained by placing the C and H atoms in the same periodic cell and in their lowest energy sites identified from the separate C and H calculations. As shown in Fig. 1, the reaction energies are obtained as ΔE = E_FS_ - E_IS_ and, as described above, the reactant and co-adsorbed product structures are used in the CI-NEB calculations to obtain the transition state structures and energies (E_TS_ in Fig. 1).

**Table 1:** ZPVE-corrected adsorption energies (eV) of all species involved in the hydrocarbon combustion reaction. Experimental data are from ref. [53] and the references for the previously calculated data are given in the footnote to the table

Species	Surface	Calculated E_ads_	Experimental E_ads_	Previous calculated E_ads_
**CH**	Ag	−3.01	–	−3.02^a^
	Au	−3.94	–	−4.19^a^
	Al	−5.67	–	–
	Cu	−4.28	–	−4.50^a^
	Rh	−5.90	–	−7.14^a^
	Pt	−6.24	–	−6.27^a^
	Pd	−5.62	–	−5.74^a^
**C**	Ag	−2.82	–	−2.27^a^
	Au	−3.82	–	−3.39^a^
	Al	−5.92	–	–
	Cu	−4.27	−5.20	−3.73^a^
	Rh	−6.44	–	−7.77^a^
	Pt	−6.44	−6.50	−6.75^a^
	Pd	−6.17	−6.94	−6.37^a^
**H**	Ag	−1.69	−2.47	−0.16^b^, 0.18^c^
	Au	−1.84	−2.52	−0.32^b^, 0.12^c^
	Al	−1.82	–	–
	Cu	−2.10	−2.43	−0.75^b^, −0.42^c^
	Rh	−2.43	−2.65	−1.89^b^, −1.55^c^
	Pt	−2.57	−2.65	−1.70^b^, −1.34^c^
	Pd	−2.47	−2.69	−2.14^b^, −1.68^c^
**C + H**	Ag	−4.17	–	–
	Au	−5.37	–	–
	Al	−7.11	–	–
	Cu	−5.97	–	–
	Rh	−8.78	–	–
	Pt	−8.58	–	–
	Pd	−8.39	–	–

^a^Ref. [54] (PW calculations using a two layer slab)

^b^Ref. [37] (GGA-PW91 calculations using a 2 × 4 unit cell and four layer slab)

^c^Ref. [37] (GGA-RPBE calculations using a 2 × 4 unit cell and four layer slab)

## Results and discussion

### Adsorption energies of CH reactants, C and H atoms, and C + H co-adsorbed products

**Fig. 3:**
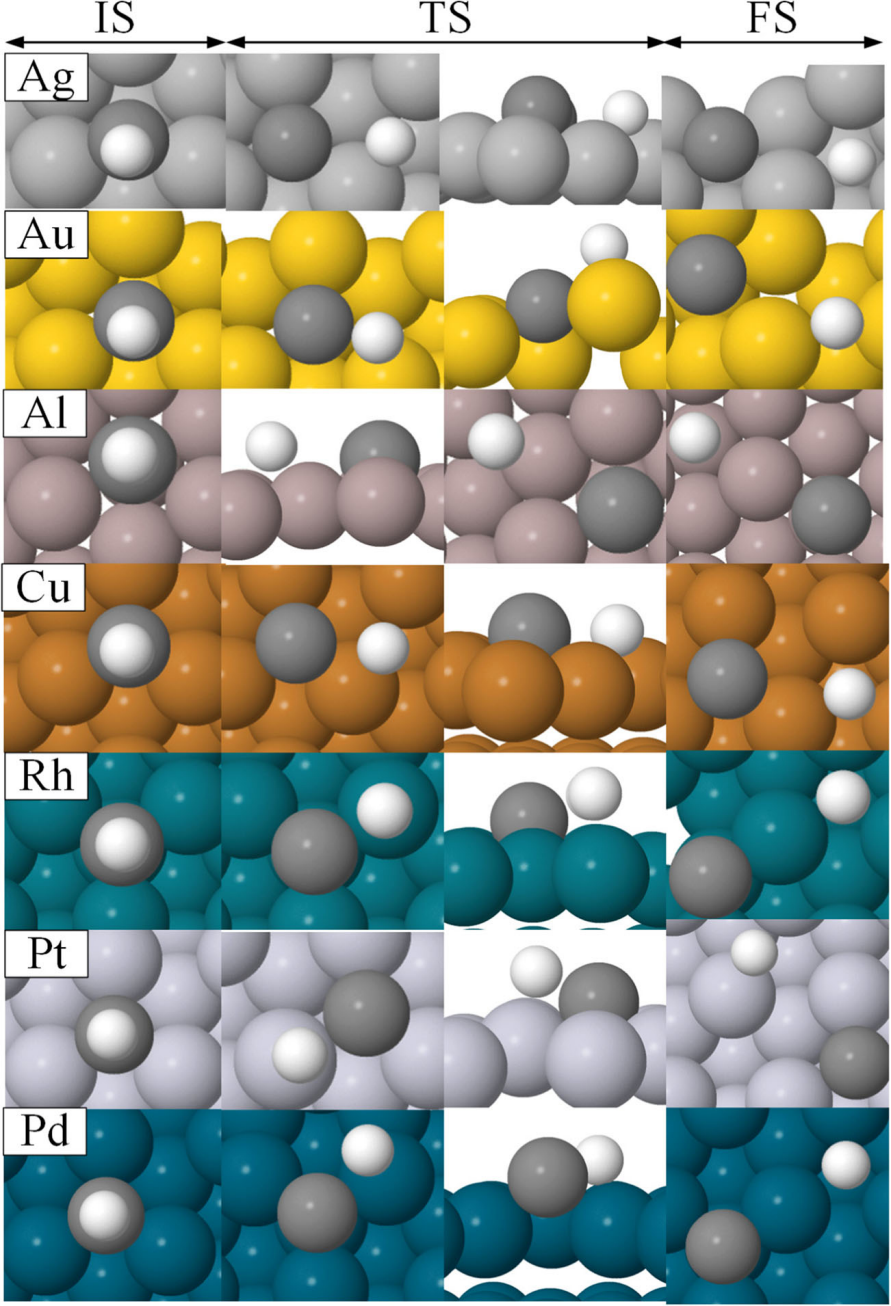
Optimized structures for the initial (IS), transition (TS), and final (FS) states for the CH → C + H reaction on surfaces

The ZPVE-corrected adsorption energies, E_ads_, for all species involved in the hydrocarbon combustion reaction (CH reactants, C and H atoms, and C + H co-adsorbed products) are presented in Table 1. Previously obtained experimental and computed data are also shown for the sake of comparison. The preferred adsorption sites, as well as data of the optimized geometries, are given in Table S1 in the SI. The optimized reactant and product geometries are also illustrated below in Fig. 3.

For CH adsorption on these surfaces, E_ads_ values range from −3.01 eV on Ag(111) to −6.24 eV on Pt (111) and decrease in the order Ag ˃ Au ˃ Cu ˃ Pd ˃ Al ˃ Rh > Pt. The calculated energies of CH adsorption are in agreement with the previous results. The trend of the CH adsorption energies obtained by Liao et al. [54] is Ag ˃ Au ˃ Cu ˃ Pd ˃ Pt > Rh, which is the same as that obtained here except for Rh and Pt, the reason for the difference between the trend in Rh and Pt is due to the larger adsorption energy of CH on Rh obtained by Liao et al. (−7.14 eV) compared to that obtained here (−5.90 eV). The average difference between the CH adsorption energies calculated here and in previous studies is 5.90%. The best agreement is for Ag and Pt, where the differences are 0.33% and 0.48%, respectively. Hence, the results presented here are in agreement with those calculated previously. The largest difference is for the Rh surface, where the difference is 21.01%. The reason for this difference could be due to the different DFT functionals used in the studies and for the different slab sizes used in the studies. No experimental results have previously been reported for CH adsorption on these metal surfaces. The preferred adsorption site for CH is the fcc site for all surfaces except for Rh, where it is the hcp site. The CH adsorption energy on the hcp site is 0.11% lower than on the fcc site for Rh, where it is −5.80 eV.

The calculated C adsorption energies decrease in the order Ag ˃ Au ˃ Cu ˃ Al ˃ Pd ˃ Rh = Pt. The strongest adsorption is −6.44 eV on the Rh (111) and Pt (111) surfaces compared to −2.82 eV on the Ag (111) surface, which is the weakest adsorption energy. The C adsorption energies in the previous study by Liao et al. follow the same trend as that discussed above for CH (Ag ˃ Au ˃ Cu ˃ Pd ˃ Pt > Rh), which is in agreement with the trend seen in the present work. Similar to the results discussed above for the CH adsorbate, the adsorption energy obtained by Liao et al. [54] on Rh (111) is −7.77 eV, which is larger than that obtained here. Our results are also in agreement with experimental observations when available. For example, the experimental adsorption energy for C on Pt (111) is −6.50 eV compare to −6.44 eV calculated here.

For H adsorption on these surfaces, E_ads_ ranges from −1.69 eV on Ag (111) to −2.57 eV on Pt (111), and decrease in the order Ag ˃ Al ˃ Au ˃ Cu ˃ Rh ˃ Pd > Pt. The most stable adsorption site for atomic hydrogen is the fcc site for all surfaces. The atomic hydrogen adsorption energies reported by Gajdos et al. [37] are lower than those reported in this study, and the difference is probably due to the fact that they used different adsorption sites, super cell size, and exchange-correlation functional. The results obtained in the present study are closer to the experimental data than those obtained previously.

The calculated (C + H) co-adsorption energies range are from −4.17 eV on the Ag (111) surface to −8.78 eV on the Rh (111), and decrease in the order Ag ˃ Au ˃ Cu ˃ Al ˃ Pd ˃ Pt > Rh. The co-adsorption energy is therefore highest on the Ag (111) surface, which was also seen for the CH, C, and H adsorbates. In all cases the co-adsorption sites are the same as those found for the isolated adsorbates (as shown in Table S1 in the Supporting information). Co-adsorption data for the products of CH splitting have not been reported previously, and hence comparison with other studies is not possible.

### Reaction and activation energies

Table 2 lists the reaction and activation energies for hydrocarbon combustion as well as the reaction rate constants at 600 K, which is a typical temperature for industrial low temperature catalytic combustion of hydrocarbons [25–27]. Data of the optimized transition state geometries as well as the imaginary frequencies of these structures are given in Table S2 in the SI. The geometries of the transition state structures are also illustrated, together with the reactant and product structures, in Fig. 3.

**Table 2:** Reaction and activation energies (eV) and reaction rate constants at 600 K (s^−1^) for the CH → C + H reaction. The energies are ZPVE-corrected

Surface		∆E(eV)	E_a_(eV)	k(s^−1^)
Ag	This work	2.20	2.43	4.77 × 10^−8^
	Previous studies	2.84^a^	1.90^a^	–
Au	This work	1.94	2.27	2.25 × 10^−6^
	Previous studies	2.71^a^	1.95^a^	–
Al	This work	1.93	2.09	3.33 × 10^−5^
	Previous studies	–	–	–
Cu	This work	1.68	1.91	9.75 × 10^−4^
	Previous studies	2.06^a^	1.86^a^	–
Rh	This work	0.49	0.98	6.79 × 10^4^
	Previous studies	0.67^b^	1.16^a^, 1.28^b^	–
Pt	This work	1.03	1.46	6.95 × 10^0^
	Previous studies	0.59^b^	1.18^a^, 1.12^b^	–
Pd	This work	0.61	1.44	3.48 × 10^1^
	Previous studies	0.72^b^	1.34^a^, 1.65^b^	–

^a^Ref. [54] (PW91 calculations using a two layer slab). Please note that some reaction energies are higher than activation energies. The reason is that the numbers reported in Ref. [54] are reaction enthalpies

^b^Ref. [55] (GGA-PBE calculations using a 2 × 2 unit cell and three layer slab)

Table 2 shows that the reaction is endothermic on all surfaces. The reaction energies for CH splitting decrease in the order Ag ˃ Au ˃ Al ˃ Cu ˃ Pt ˃ Pd > Rh. Similar reaction energies are reported in the previous studies. Inderwildi et al. [55] found endothermic reaction energies on the Rh (111), Pt (111), and Pd (111) surfaces of 0.67 eV, 0.59 eV, and 0.72 eV, respectively. These are similar to the energies obtained in the present study, except for the Pt surface where the previously calculated value is less than the value obtained here (1.03 eV). The reason for this may be the different functionals used in the two studies. On average the reaction energies from the present calculations and those obtained by Inderwildi et al. differ by −4.20%. The reaction energies obtained here are not in as good agreement with those obtained by Liao et al. [54], where a smaller, two layer slab was used.

The results in Table 2 show that the activation energies for dissociation of CH decrease in the order Ag ˃ Au ˃ Al ˃ Cu ˃ Pt ˃ Pd ˃ Rh. The lowest activation energy for CH splitting, obtained on the Rh (111) surface, was 0.98 eV, compared to 2.43 eV on Ag (111). Thus, Ag is the least active catalyst. The trend observed for the activation energies for these metal surfaces is discussed in more details in Physical properties that control the relative adsorption energies of adsorbates on metal surfaces. Similar activation energies are obtained for Pt (111) and Pd (111), 1.46 eV and 1.44 eV respectively. Liao et al. [54] obtained lower activation barriers for CH dissociation on all surfaces except for Rh (111) surface, where they obtained a higher activation energy of 1.16 eV, compared to 0.98 eV obtained here. Inderwildi et al. [55] obtained activation energies of 1.28 eV, 1.12 eV, and 1.65 eV on the Rh (111), Pt (111), and Pd (111) surfaces, respectively, which are similar to the values obtained in this work of 0.98 eV, 1.46 eV, and 1.44 eV. As discussed above, these differences may be due to difference in the functionals and system sizes used in the studies.

The reaction rate constants are strongly influenced by the different metal surfaces, and at 600 K they decrease from 6.79 × 10^4^ s^−1^ on Rh (111) to 4.77 × 10^−8^ s^−1^ on Ag (111). It is clear that the trend in rate constants is very similar to the trend in activation energies, with a higher activation energy leading to a lower rate constant. Rate constants of CH splitting on the surfaces studied here have not previously been reported, and hence comparison cannot be made with previous studies.

### Brønsted-Evans-Polanyi relationships

**Fig. 4:**
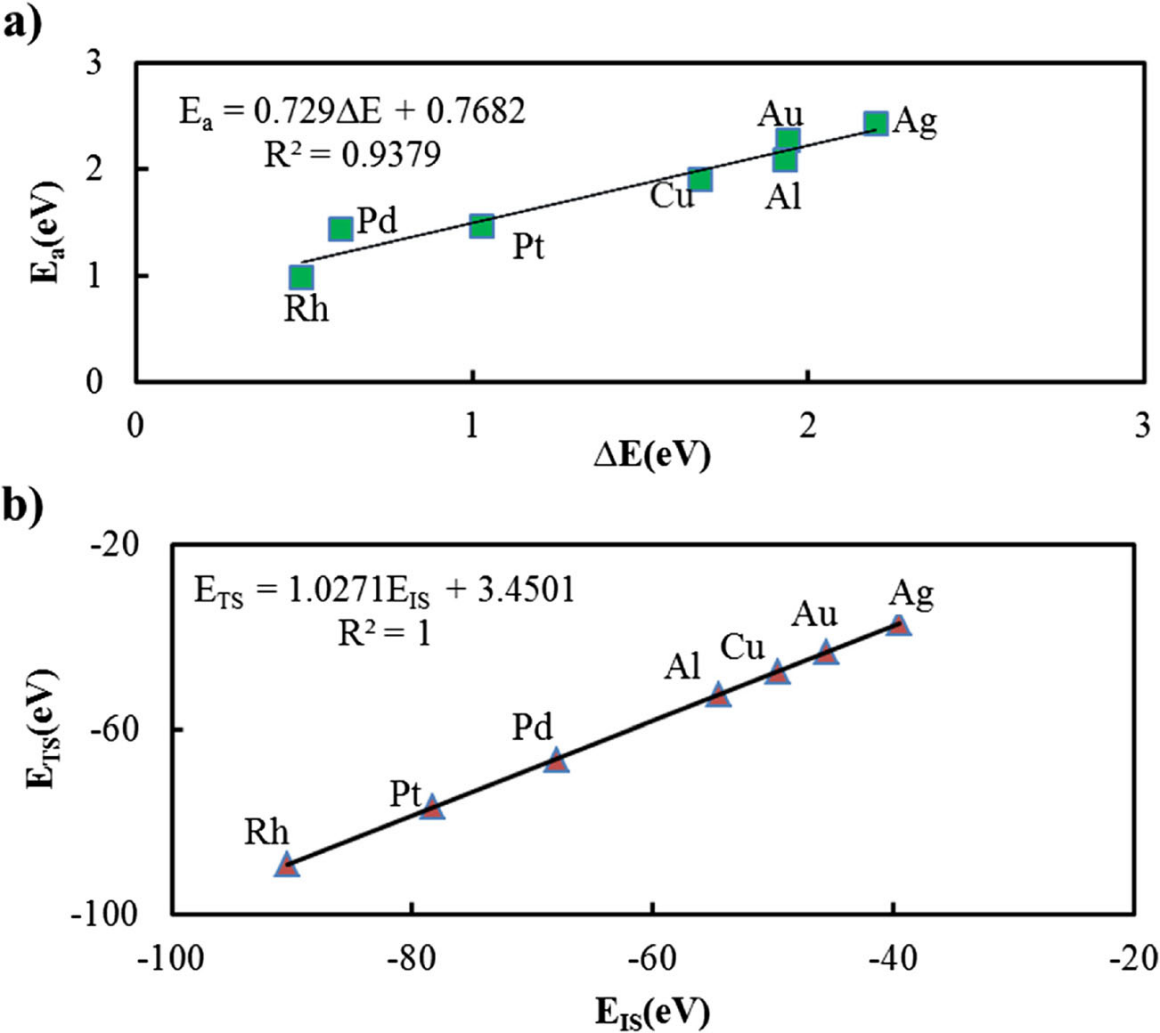
(a) BEP and (b) TSS relations for the CH → C + H dissociation on the metallic surfaces listed in Table 1

The BEP and TSS relations for the hydrocarbon combustion reaction on the metal surfaces listed in Table 1 are presented in Fig. 4. It can be seen that BEP relation is a very good descriptor for this reaction on these metal surfaces, with an R^2^ value of 0.94. The TSS relation is an excellent descriptor with an R^2^ value of 1.

Since the BEP and TSS relations are very good descriptors for CH → C + H dissociation on the metal surfaces listed in Table 1, they were used to estimate the activation energy, E_a_, and transition state energy, E_TS_, for this reaction on Ni (face-centered-cubic (111) surface), Co (hexagonal-closed-packed (111) surface), and Fe (body-centered-cubic (111) surface) surfaces. Hence, the methods described in Methods and models were used to calculate the adsorption energies for the CH reactant and co-adsorbed C + H product for these metal surfaces, which are shown in Table S3 in the SI.

The reaction energies, ∆E, were determined from the ZPVE-corrected reactant and product energies, and are 0.59, 0.43, and 0.09 for the Ni, Co, and Fe surfaces, respectively. These values were used in the BEP relation shown in Fig. 4a (E_a_ = 0.729 ∆E + 0.7682) to determine the BEP-predicted activation energies, which are shown in Table 3. Similarly, the TSS relation in Fig. 4b (E_TS_ = 1.0271 E_IS_ + 3.4501) was used to calculate the TSS-predicted transition state energies shown in Table 3.

**Table 3:** BEP-predicted E_a_, TSS-predicted E_TS_, and the corresponding energies explicitly calculated from DFT using the methods described in Methods and models. All energies are in eV

Surface	BEP-predicted E_a_ (eV)	TSS-predicted E_TS_ (eV)	DFT-calculated E_a_ (eV)	DFT-calculated E_TS_ (eV)
**Ni**	1.20	−69.70	1.23	−69.98
**Co**	1.08	−87.93	1.08	−87.88
**Fe**	0.83	−92.45	0.79	−92.57

**Fig. 5:**
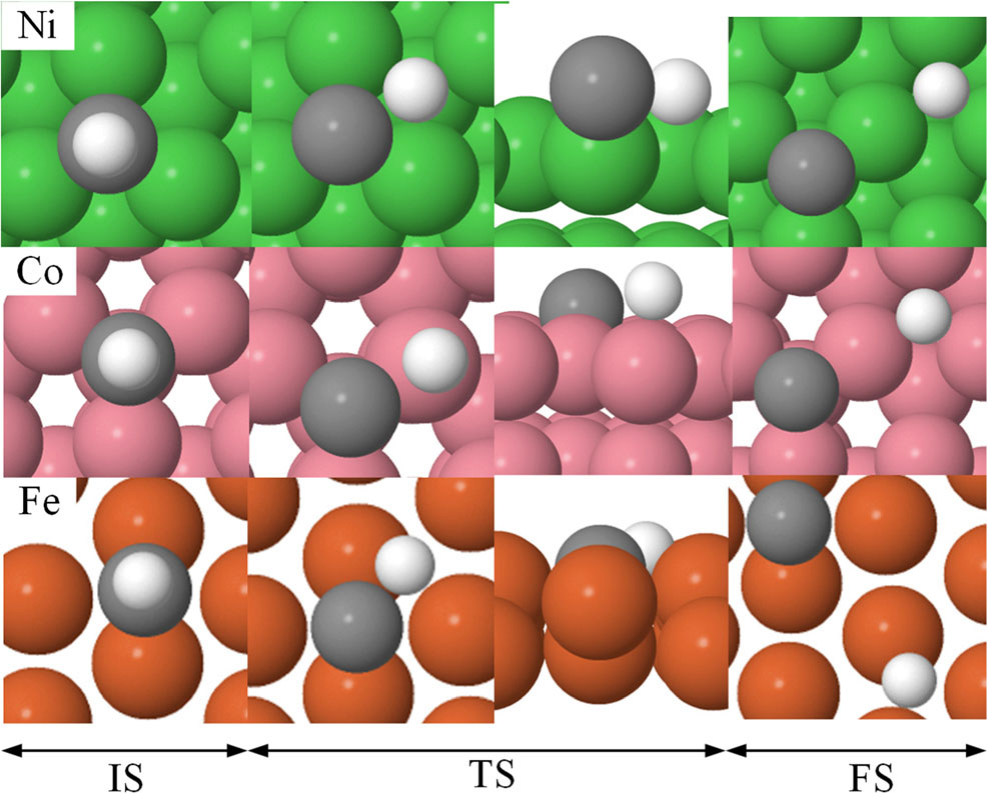
Optimized structures for the initial (IS), transition (TS), and final (FS) states for the CH → C + H reaction on the Ni, Co, and Fe (111) surfaces

The same computational methods discussed in Methods and models were then used to ascertain the accuracy of the predicted energies. The activation and transition state energies that were obtained from the DFT calculations are shown in columns 4 and 5 in Table 3. Additional data of the transition state structures are given in Table S4 in the SI, and they are illustrated in Fig. 5.

The activation and transition state energies estimated by the BEP and TSS correlations on Ni, Co, and Fe surfaces are in excellent agreement with those obtained by the DFT calculations (Table 3). In fact, the average difference in the DFT-calculated E_a_ and the BEP-predicted E_a_ is 0.15%, where the best agreement is for Co with error value 0.15%. Similarly, there is only a 0.04% average difference in DFT-calculated E_TS_ and TSS-predicted E_TS_. Therefore, these correlations can be used to efficiently predict energetics of similar reactions on these surfaces without doing time consuming transition state calculations.

It should be noted that, considering the obtained R^2^ values, the TSS theory appears to have a better fit to the DFT results. However, the values shown in Table 3 show that BEP would provide a better prediction of the barrier height. Another method that can be used to evaluate the accuracy of the linear BEP and TSS relations is cross validation (CV). This method is primarily used to evaluate the predictive validity of linear regression equations [56] where, in the present work, the DFT and predicted energies of the Ni, Co, and Fe systems comprise the test set and the energies from the other seven metal systems comprise the training set. The calculated CV statistics for the BEP and TSS models (0.001 for BEP and 0.033 for TSS) show that the BEP descriptor is expected to perform better than TSS. In spite of this, the TSS relation may be preferred since the structure of either the initial or final state, and not both, is sufficient for estimating the activation barrier.

For the sake of completeness, the BEP and TSS relations using all of the metal surfaces are given in Fig. S1 in the SI. An R^2^ value of 0.97 (compared to 0.94 when Ni, Co, and Fe were omitted) was obtained for the BEP relation and 0.99 (compared to 1.0) for the TSS relation. This is further indication that the BEP and TSS relations are good predictors for hydrocarbon combustion on these surfaces.

Prediction of the activation energies or rate constants could be further simplified if it was based on only the reactant or product adsorption energies instead of the reaction energy (which requires calculation of both energies). This has been suggested earlier for prediction of rate constants by Fajin et al. [22] who found a significant dependence of the calculated rate constants on the E_ads_ of selected species. These new types of relationships allow for a direct estimation of the reaction rate constant (for water splitting in the previous study [22]) from the adsorption energy of an adsorbate (atomic oxygen in their study [22]). Comparison of panels a and b in Fig. S2 in the SI shows that, for the CH → C + H reaction on these metals surfaces, it is more accurate to use the product adsorption energies (R^2^ = 0.88) than the reactant adsorption energies (R^2^ = 0.72) to predict the reaction rate constants. However, it should be noted that the log of the reaction rate constants is used in Fig. S2, which means that predicting the actual rate constant is even more uncertain.

### Physical properties that control the relative adsorption energies of adsorbates on metal surfaces

As discussed with reference to Fig. 1, the thermodynamics of any reaction is determined by the relative energies of the products and reactants. The kinetics is also affected by the relative energies of the transition states and the reactants (forward reaction) and products (reverse reaction). It is therefore relevant to try to understand the physical properties that determine these energies.

As discussed above, the adsorption energies of CH decrease in the order Ag ˃ Au ˃ Cu ˃ Pd ˃ Co ˃ Al > Ni ˃ Rh ˃ Pt > Fe, for C in the order Ag ˃ Au ˃ Cu ˃ Al ˃ Ni ˃ Co = Pd ˃ Rh = Pt ˃ Fe, for H in the order Ag ˃ Al ˃ Au ˃ Cu ˃ Co ˃ Rh ˃ Ni ˃ Pd ˃ Pt > Fe, and for C + H in the order Ag ˃ Au ˃ Cu ˃ Al ˃ Pd ˃ Ni ˃ Pt ˃ Co ˃ Rh ˃ Fe. Hence, these trends are similar. For all adsorbates, the weakest adsorption is on the Ag (111), Au (111), and Cu (111) surfaces, respectively; whereas the strongest adsorption is on the Fe (111) surface. Also, the activation energies for CH dissociation decrease in the order Ag ˃ Au ˃ Al ˃ Cu ˃ Pt ˃ Pd ˃ Rh.

A common way to try to understand trends in adsorption and activation energies is based on the d-electrons of the metal surface [22, 57, 58]. It is argued that these d-electrons can be transferred to the adsorbate, hence increasing the binding energy, if they are nearer to the Fermi level (this argument may be relevant to the present studies since earlier calculations have shown that the adsorbates gain negative charge when they are adsorbed on the surface [52, 59]). The d-band center is usually used to measure the proximity of the d-electrons to the Fermi level, [10, 60] and is calculated from the projected density of states (PDOS) of the d-orbitals. However, it should be noted that the d-band model is usually used to compare trends in adsorption and activation energies of a given adsorbate on different surface structures of the same metal surface, and not on surfaces of different metals, as is the case here [61].

In spite of this, the PDOS was calculated for the top atomic layer of the metal surfaces studied here, and the results are shown in Fig. S3 in the SI. Based on Fig. S3 one would expect the adsorption energies to decrease in the order Au ˃ Ag ˃ Cu ˃ Pd ˃ Pt ˃ Rh. This is different from the order of the adsorption and activation energies of CH, C, H, C + H, and the transition states given above, and hence does not explain the trends seen in this work. These results are similar to those obtained by Fajin et al. who reported that the d-band model cannot be used to understand the origin of the BEP relationship of water splitting on different metallic surfaces [22].

The coordination number of the metal surface atoms is also used to explain trends in adsorption and activation energies [62–64]. This is not relevant for the present work since the coordination numbers for all (111) surfaces studied here are identical. Further work must therefore focus on improved understanding of the geometric and electronic features that determine the thermodynamic and kinetic trends of the hydrocarbon combustion reaction on different metal surfaces.

## Conclusions

Spin polarized, GGA DFT calculations using the RPBE functional and the PAW method were used to explore the thermodynamic and catalytic activity of various metal surfaces for hydrocarbon combustion (CH → C + H), as well as the relevance of the BEP and TSS relations for this reaction. Data obtained for the Ag, Au, Al, Cu, Rh, Pt, and Pd surfaces revealed that these relations are valid (R^2^ = 0.94 for the BEP correlation and R^2^ = 1.0 for the TSS correlation) for these systems. They were therefore used to estimate the energetics of the combustion reaction on Ni, Co, and Fe surfaces. The estimated transition state and activation energies (E_TS_ = −69.70 eV and E_a_ = 1.20 eV for Ni, E_TS_ = −87.93 eV and E_a_ = 1.08 eV for Co and E_TS_ = −92.45 eV and E_a_ = 0.83 eV for Fe) are in agreement with those obtained by DFT calculations (E_TS_ = −69.98 eV and E_a_ = 1.23 eV for Ni, E_TS_ = −87.88 eV and E_a_ = 1.08 eV for Co and E_TS_ = −92.57 eV and E_a_ = 0.79 eV for Fe). Therefore, these relations can be used to predict energetics of this reaction on these surfaces without doing the time consuming transition state calculations.

Also, the calculations show that the activation energy for CH → C + H decreases in the order Ag ˃ Au ˃ Al ˃ Cu ˃ Pt ˃ Pd ˃ Ni > Co > Rh > Fe. This, combined with the Arrhenius pre-exponential, revealed that, among the different surfaces considered in this work, Fe is the best catalyst for the CH → C + H reaction while Ag is the least active surface.

## Electronic supplementary material

ESM 1 (DOCX 6021 kb)
