# Supplementary material for: DFT studies of hydrocarbon combustion on metal surfaces
Source: J Mol Model. 2018 Feb 2;24(2):47. doi: 10.1007/s00894-018-3585-z (PMC5797216; doi:10.1007/s00894-018-3585-z)
Supplement: Supplementary file 1 — (DOCX 6021 kb) [file 894_2018_3585_MOESM1_ESM.docx]

**Supporting information**

**DFT studies of hydrocarbon combustion on metal surfaces**

Journal of Molecular Modeling

Mina Arya^1,2,*^, Ali Akbar Mirzaei^2^, Abdol Mahmood Davarpanah^3^, Seyed Masoud Barakati^4^, Hossein Atashi^5^ , Abas Mohsenzadeh^1^, Kim Bolton^1^

^1^Swedish Centre for Resource Recovery, University of Borås, SE 501-90 Borås, Sweden

^2^Department of Chemistry, University of Sistan and Baluchestan, Zahedan 98135-674, Iran

^3^Department of Physics, University of Sistan and Baluchestan, Zahedan 98135-674, Iran

^4^Department of Electrical and Computer Engineering, University of Sistan and Baluchestan, Zahedan 98135-674, Iran

^5^Department of Chemical Engineering, University of Sistan and Baluchestan, Zahedan 98135-674, Iran

E-Mail Addresses: [mina.arya@hb.se](mailto:mina.arya@hb.se) (M. Arya); [mirzaei@hamoon.usb.ac.ir](mailto:mirzaei@hamoon.usb.ac.ir) (A.A. Mirzaei); [a.m.davarpanah@phys.usb.ac.ir](mailto:mirzaei@hamoon.usb.ac.ir) (A.M. Davarpanah); [smbaraka@ece.usb.ac.ir](mailto:smbaraka@ece.usb.ac.ir) (S.M. Barakati); [h.ateshy@hamoon.usb.ac.ir](mailto:h.ateshy@hamoon.usb.ac.ir) (H. Atashi); abas.mohsenzadeh@hb.se (A. Mohsenzadeh); kim.bolton@hb.se (K. Bolton)

^*^Corresponding author: E-mail: [mina.arya@hb.se](mailto:mina.arya@hb.se) (M. Arya); Tel.:+46-33-4354534; Fax: +46-33-4354008; Address: University of Sistan and Baluchestan, Zahedan 98135-674, Iran

**Table S1** Adsorption energies (eV) and structural parameters (Å) of the chemical species involved in CH dissociation to C and H, the adsorption energies are ZPVE-corrected values. d_surf-C_ and d_surf-H_ are the shortest distances between carbon and hydrogen atoms of the adsorbate and any metal atom on the surface. d_C-H_ is the distance between H and C atoms

| **Species** | **Surface** | **Most stable site** | **E_ads_(eV)** | **d_surf-C_(Å)** | **d_surf-H_(Å)** | **d_C-H_(Å)** |
| --- | --- | --- | --- | --- | --- | --- |
| **CH** | Ag | fcc | -3.01 | 2.117 | 2.919 | 1.103 |
|  | Au | fcc | -3.94 | 2.034 | 2.745 | 1.099 |
|  | Al | fcc | -5.67 | 1.982 | 2.714 | 1.102 |
|  | Cu | fcc | -4.28 | 1.907 | 2.722 | 1.101 |
|  | Rh | hcp | -5.90 | 1.983 | 2.791 | 1.103 |
|  | Pt | fcc | -6.24 | 2.013 | 2.806 | 1.099 |
|  | Pd | fcc | -5.62 | 1.972 | 2.768 | 1.105 |
|  | Ni | fcc | -5.71 | 1.850 | 2.683 | 1.102 |
|  | Co | hcp | -5.66 | 1.892 | 2.691 | 1.105 |
|  | Fe | long bridge | -7.70 | 2.070 | 2.630 | 1.106 |
| **C + H** | Ag | C: fcc | -4.17 | 2.099 | 1.842 | 2.920 |
|  |  | H: fcc |  |  |  |  |
|  | Au | C: fcc | -5.37 | 1.983 | 1.752 | 3.112 |
|  |  | H: fcc |  |  |  |  |
|  | Al | C: hcp | -7.11 | 1.933 | 1.876 | 3.292 |
|  |  | H: fcc |  |  |  |  |
|  | Cu | C: fcc | -5.97 | 1.878 | 1.677 | 2.573 |
|  |  | H: fcc |  |  |  |  |
|  | Rh | C: hcp | -8.78 | 1.903 | 1.839 | 4.056 |
|  |  | H: fcc |  |  |  |  |
|  | Pt | C: fcc | -8.58 | 1.926 | 1.843 | 3.264 |
|  |  | H: fcc |  |  |  |  |
|  | Pd | C: hcp | -8.39 | 1.918 | 1.737 | 3.305 |
|  |  | H: fcc |  |  |  |  |
|  | Ni | C: hcp | -8.49 | 1.785 | 1.694 | 2.939 |
|  |  | H: fcc |  |  |  |  |
|  | Co | C: hcp | -8.60 | 1.799 | 1.687 | 3.847 |
|  |  | H: fcc |  |  |  |  |
|  | Fe | C: long bridge | -10.98 | 1.832 | 1.803 | 3.936 |
|  |  | H: long bridge |  |  |  |  |

**Table S2** Most stable sites, structural parameters (Å) and imaginary frequencies (cm^-1^) of the transition states in CH dissociation to C and H. The adsorption energies are ZPVE-corrected values. d_surf-C_ and d_surf-H_ are the shortest distances between carbon and hydrogen atoms of the adsorbate and any metal atom on the surface. d_C-H_ is the distance between C and H atoms

| **Surface** | **Most stable site** | **d_surf-C_(Å)** | **d_surf-H_(Å)** | **d_C-H_(Å)** | **Imaginary frequency(cm^-1^)** |
| --- | --- | --- | --- | --- | --- |
| Ag | C: fcc | 2.128 | 1.778 | 2.098 | 689 |
|  | H: bridge |  |  |  |  |
| Au | C: fcc | 2.009 | 1.623 | 1.976 | 724 |
|  | H: bridge |  |  |  |  |
| Al | C: fcc | 1.938 | 2.500 | 2.713 | 356 |
|  | H: top |  |  |  |  |
| Cu | C: fcc | 1.880 | 1.624 | 1.819 | 627 |
|  | H: bridge |  |  |  |  |
| Rh | C: hcp | 1.901 | 3.139 | 1.662 | 798 |
|  | H: top |  |  |  |  |
| Pt | C: fcc | 1.912 | 1.616 | 1.676 | 848 |
|  | H: top |  |  |  |  |
| Pd | C: hcp | 1.901 | 1.689 | 1.612 | 994 |
|  | H: fcc |  |  |  |  |

**Table S3** Adsorption energies (eV) of the chemical species involved in CH dissociation to C and H. The adsorption energies are ZPVE-corrected values

| **Species** | **Surface** | **Most stable site** | **Calculated E_ads_(eV)** | **Experimental E_ads_(eV)** | **Previous calculated E_ads_(eV)** |
| --- | --- | --- | --- | --- | --- |
| **CH** | Ni | fcc | -5.71 | -5.70^a^, -5.89^b^ | -6.25^d^ |
|  | Co | hcp | -5.66 | - | - |
|  | Fe | long bridge | -7.70 | - | - |
| **C** | Ni | hcp | -6.10 | -7.42^c^ | -7.65^e^ |
|  | Co | hcp | -6.17 | - | - |
|  | Fe | long bridge | -8.58 | - | - |
| **H** | Ni | fcc | -2.45 | -2.73^c^ | -2.66^f^, -2.78^g^, -2.77^h^, -2.80^i^, -0.60^j^ |
|  | Co | fcc | -2.42 | - | -1.65^k^, -1.32^l^ |
|  | Fe | long bridge | -4.36 | - | - |
| **C + H** | Ni | C: hcp | -8.49 | - | - |
|  |  | H: fcc |  | - | - |
|  | Co | C: hcp | -8.60 | - | - |
|  |  | H: fcc |  | - | - |
|  | Fe | C: long bridge | -10.98 | - | - |
|  |  | H: long bridge |  | - | - |

^a^  Ref. [1].

^b^  Ref. [2].

^c^  Ref. [3].

^d^  Ref. [4] (GGA-PBE calculations using a 2×2 unit cell and four layer slab)

^e^  Ref. [5] (PW91 calculations using a two layer slab)

^f^  Ref. [6] (GGA-PBE calculations using a 2×2 unit cell and four layer slab)

^g^ Ref. [7] (GGA-PBE calculations using a 2×2 unit cell and three layer slab)

^h^ Ref. [8] (GGA-PBE calculations using a 2×2 unit cell and four layer slab)

^i^ Ref. [9] (GGA-PBE calculations using a 2×2 unit cell and four layer slab)

^j^ Ref. [10] (GGA-PW91 calculations using a 2×2 unit cell and four layer slab)

^k^ Ref. [11] (GGA-PW91 calculations using a 2×4 unit cell and four layer slab)

^l^ Ref. [11] (GGA-RPBE calculations using a 2×4 unit cell and four layer slab)

For CH adsorption on these surfaces, the strongest adsorption is -7.70 eV on the Fe (111) surface, compared to -5.71 eV for Ni (111) and -5.66 eV for Co (111). Experimental investigations also support our calculated adsorption energies. According to the experimental data, the adsorption energies are -5.70 eV, -5.89 eV on the Ni (111) surface, that are in agreement with those obtained here.

The calculated C adsorption energy follows the trend Fe < Co < Ni. The strongest adsorption is on the Fe (111) and is -8.58 eV. This can be compared to -6.17 eV for Co (111) and -6.10 eV for Ni (111). The results presented here are in agreement with those obtained previously. For example, Fajin et al. (using PW91 calculations) obtained adsorption energy of -7.65 eV for Ni (111). Our results are also supported by experimental observations when available. For example the experimental adsorption energy for Ni (111) is -7.42 eV compare to -6.10 eV, thus showing agreement with our results.

The H adsorption energy follows the same trend as that discussed above for CH and E_ads_ values range from -4.36 eV on Fe (111) to -2.42 eV on Co (111). Mohsenzadeh et al. obtained an adsorption energy of -2.66 eV for H on the Ni (111) surface. Gajdoš et al. calculated an adsorption energy of -1.65 eV on the Co (111) surface. Hence the results obtained in the present work are in agreement with previously calculated data when available. Experimental investigations also support our results. According to the experimental data, the adsorption energy is -2.73 eV on the Ni (111) surface, then that are in very good agreement with the obtained here.

The calculated co-adsorption energies follow the same trend as that discussed above for C. The strongest adsorption is -10.98 eV on the Fe (111) surface, compared to -8.60 eV for Co (111) and -8.49 eV for Ni (111). Co-adsorption data for the products of CH splitting have not been reported previously, and hence comparison with other studies is not possible.

**Table S4** Most stable sites, structural parameters (Å) and imaginary frequencies (cm^-1^) of the transition states in CH dissociation to C and H on the Ni, Co and Fe (111) surfaces. The adsorption energies are ZPVE-corrected values. d_surf-C_ and d_surf-H_ are the shortest distances between carbon and hydrogen atoms of the adsorbate and any metal atom on the surface. d_C-H_ is the distance between H and C atoms

| **Surface** | **Most stable site** | **d_surf-C_(Å)** | **d_surf-H_(Å)** | **d_C-H_(Å)** | **Imaginary frequency(cm^-1^)** |
| --- | --- | --- | --- | --- | --- |
| Ni | C: fcc | 1.826 | 1.775 | 1.446 | 781 |
|  | H: hcp |  |  |  |  |
| Co | C: hcp | 1.807 | 1.521 | 1.670 | 847 |
|  | H: top |  |  |  |  |
| Fe | C: long bridge | 1.843 | 1.740 | 1.463 | 891 |
|  | H: long bridge |  |  |  |  |


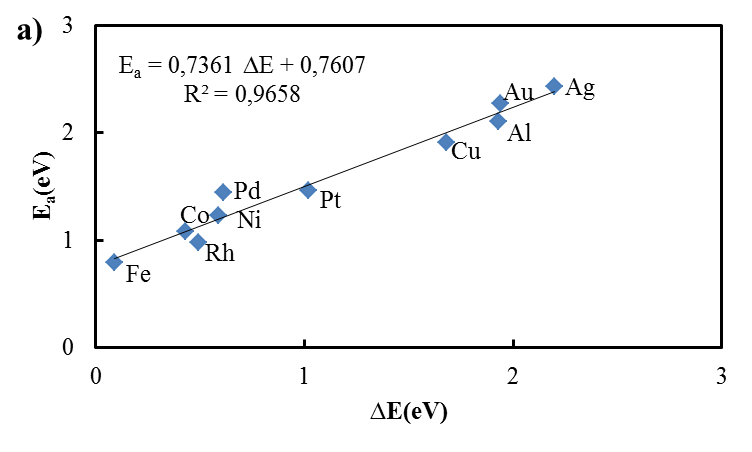


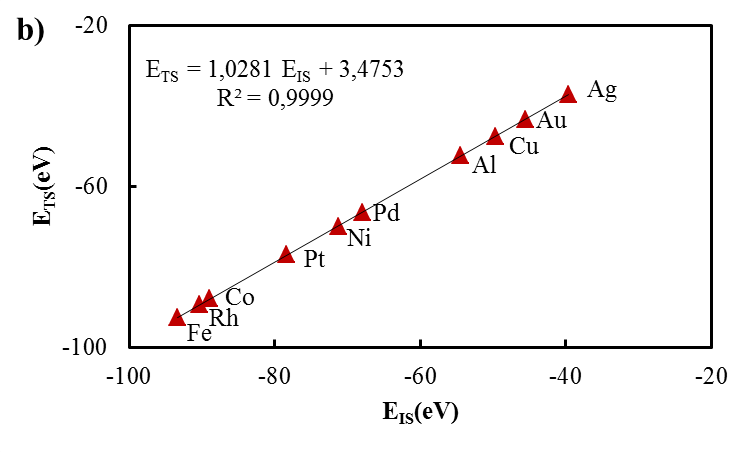


**Fig. S1** Relationships between (a) activation energy barrier and reaction energy (BEP relation), (b) initial state energy and transition state energy (TSS correlations), for the CH → C + H reaction on the metal surfaces listed in Table S1


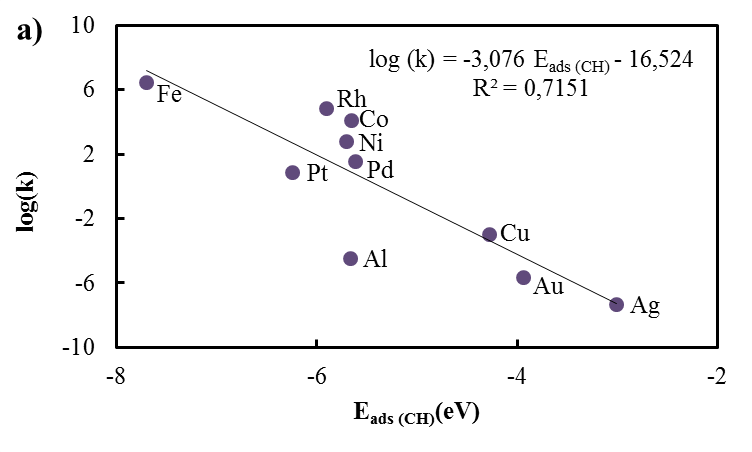


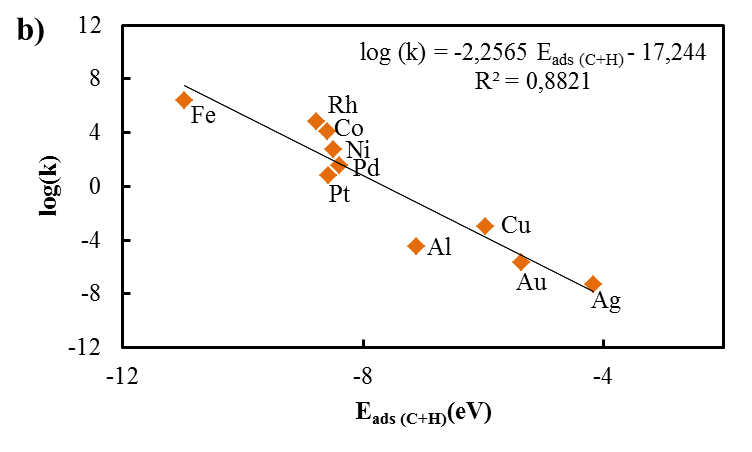


**Fig. S2** Relationships between the logarithm of the rate constant (log k) and (a) adsorption energy of CH and (b) co-adsorption energy of C and H species

**
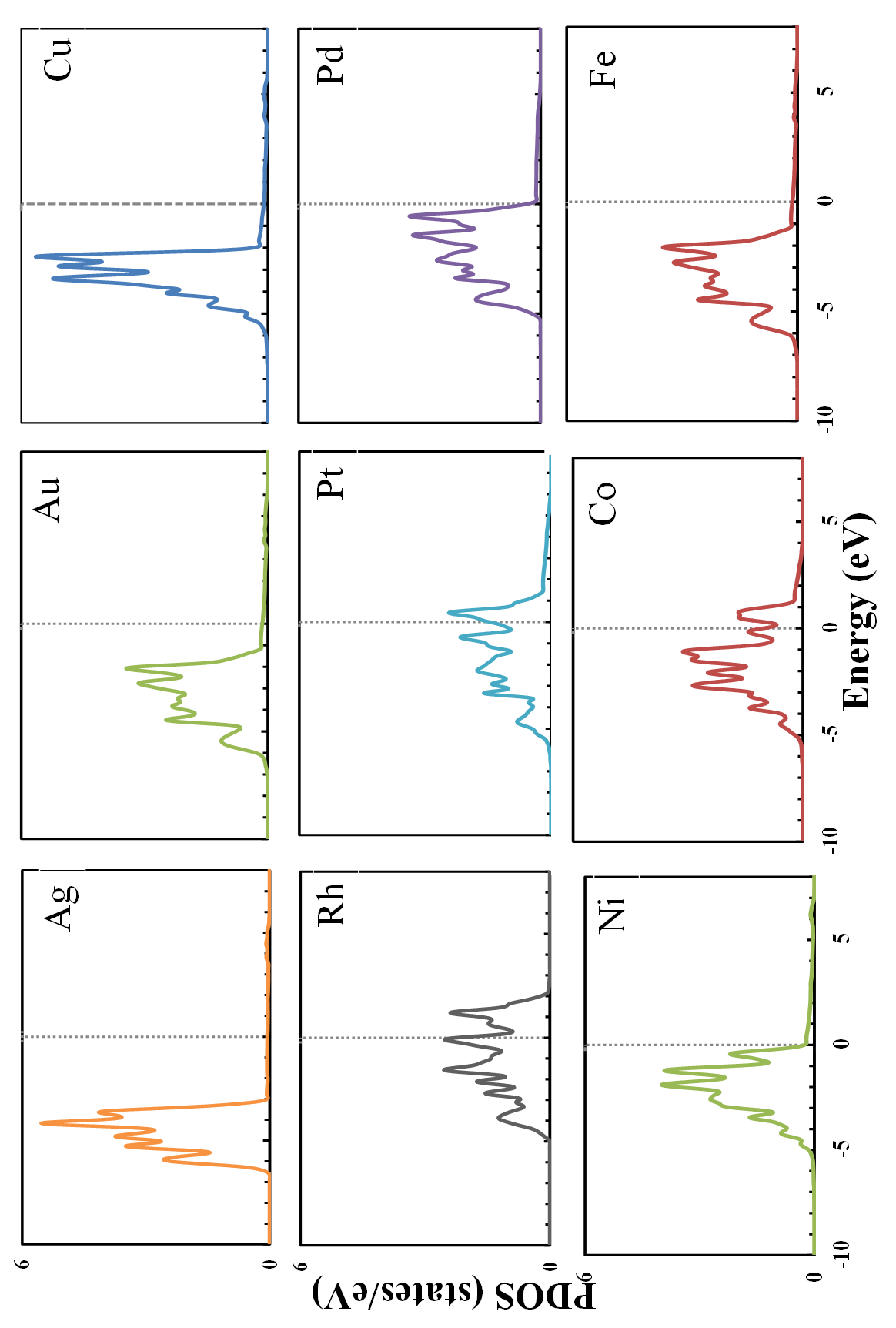
**

**Fig. S3** Projected density of states (PDOS) plots of the d-orbitals of the uppermost atoms on Ag(111), Au(111), Cu(111), Rh(111), Pt(111), Pd(111), Fe(111), Ni(111) and Co(111) surfaces

**References**

1. Li J, Croiset E, Ricardez-Sandoval L (2012) Methane dissociation on Ni (100), Ni (111), and Ni (553): A comparative density functional theory study. Journal of Molecular Catalysis A: Chemical 365:103-114

2. Siegbahn PEM, Panas I (1990) A theoretical study of CH_x_ chemisorption on the Ni (100) and Ni (111) surfaces. Surface science 240 (1-3):37-49

3. Au C-T, Ng C-F, Liao M-S (1999) Methane Dissociation and Syngas Formation on Ru, Os, Rh, Ir, Pd, Pt, Cu, Ag, and Au: A Theoretical Study. Journal of Catalysis 185 (1):12-22

4. Mohsenzadeh A, Richards T, Bolton K (2015) A density functional theory study of hydrocarbon combustion and synthesis on Ni surfaces. Journal of molecular modeling 21 (3):46

5. Liao M-S, Zhang Q-E (1998) Dissociation of methane on different transition metals. Journal of Molecular Catalysis A: Chemical 136 (2):185-194

6. Mohsenzadeh A, Bolton K, Richards T (2014) DFT study of the adsorption and dissociation of water on Ni (111), Ni (110) and Ni (100) surfaces. Surface Science 627:1-10

7. Pozzo M, Carlini G, Rosei R, Alfè D (2007) Comparative study of water dissociation on Rh (111) and Ni (111) studied with first principles calculations. The Journal of chemical physics 126 (16):164706

8. Catapan RC, Oliveira AAM, Chen Y, Vlachos DG (2012) DFT study of the water–gas shift reaction and coke formation on Ni (111) and Ni (211) surfaces. The Journal of Physical Chemistry C 116 (38):20281-20291

9. Seenivasan H, Tiwari AK (2013) Water dissociation on Ni (100) and Ni (111): Effect of surface temperature on reactivity. The Journal of chemical physics 139 (17):174707

10. Kresse G, Hafner J (2000) First-principles study of the adsorption of atomic H on Ni (111),(100) and (110). Surface science 459 (3):287-302

11. Gajdoš M, Eichler A, Hafner J (2004) CO adsorption on close-packed transition and noble metal surfaces: trends from ab initio calculations. Journal of Physics: Condensed Matter 16 (8):1141
